# Evidence-based AI: from trailblazer to trustblazer?

**DOI:** 10.3389/frai.2026.1818128

**Published:** 2026-06-03

**Authors:** Thomas Luechtefeld, Thomas Hartung

**Affiliations:** 1 Insilica Inc., Rockville, MD, United States; 2 Center for Alternatives to Animal Testing (CAAT), Johns Hopkins Bloomberg School of Public Health, Baltimore, MD, United States; 3 CAAT-Europe, University of Konstanz, Konstanz, Germany

**Keywords:** agentic AI, e-validation, evidence-based edicine, evidence-based toxicology, regulatory science, retrieval-augmented generation, risk of bias, systematic review

## Abstract

Agentic AI systems can plan, call tools, and coordinate specialized sub-agents, enabling multi-step scientific workflows that exceed what single-model text generation can reliably deliver. Yet in high-stakes domains such as regulatory science and toxicology, fluent outputs are not sufficient: adoption hinges on traceability, reproducibility, context-of-use validity, and explicit uncertainty communication. This perspective argues that evidence-based medicine and evidence-based toxicology provide a mature epistemic scaffold for making agentic AI trustworthy by design. We propose an Evidence-based Agent Stack that decomposes end-to-end tasks into protocolized roles (question framing, retrieval, screening, extraction, risk-of-bias appraisal, synthesis, mechanistic/causal integration, uncertainty assessment, and evidence-to-decision translation) with mandatory provenance and versioning. Anchoring agentic workflows in systematic review practice, risk-of-bias frameworks, and emerging regulatory principles (e.g., TREAT and e-validation) can turn “*trailblazing*” AI into “*trustblazing*” AI: systems whose outputs are auditable, updateable, and aligned with decision accountability.

## 1 Introduction: why trust is now the limiting reagent

Generative AI has become a catalytic technology for scientific work, accelerating drafting, coding, literature triage, and hypothesis generation, but fluency is an unreliable proxy for truth. Large language models are trained to produce *likely* continuations of text, not to guarantee factuality, and they can generate plausible statements that are ungrounded or subtly wrong (“*hallucinations*”). This is not a theoretical corner case: formal evaluations show that scaling and polish do not necessarily improve truthfulness, especially on questions that elicit common misconceptions (Lin et al., 2022), while broader analyses have emphasized how opaque training data, documentation gaps, and externalities can undermine scientific and societal trust in large models even when outputs look authoritative (Bender et al., 2021; Huang et al., 2023).

Agentic AI raises the stakes (Luechtefeld and Hartung, 2025) because it turns a model from a “*text generator*” into an actor capable of *long action chains*: a system can retrieve evidence, screen and extract data, summarize, draft conclusions, populate decision tables, and trigger downstream actions. These multi-step loops make productivity gains real, but also create new failure modes: small upstream errors can propagate, compounding into confident downstream recommendations, and the chain itself can obscure where uncertainty entered. The current wave of “*reason-and-act*” and tool-using paradigms makes this explicit: agents interleave reasoning with external actions (e.g., search, database queries, calculations), and increasingly coordinate in multi-agent workflows (Yao et al., 2022; Schick et al., 2023; Wu et al., 2023). In such systems, the epistemic risk is not only *a wrong answer*; it is a wrong workflow that appears internally coherent.

In regulatory contexts, the emerging consensus is that trust is essential yet difficult to operationalize, and even “*classic*” scientific desired aspects such as reproducibility and explainability are being renegotiated for generative systems. A central tension, raised directly in recent regulatory-science discussions, is that different models may use different internal features yet achieve similar performance; which, then, should be trusted, and what does “*reproducible*” mean when outputs can vary stochastically across runs (Hartung et al., 2025a)? At the same time, the broader reproducibility debate in AI has underscored how incomplete reporting, unavailable code/data, and under-specified pipelines can make apparently strong results fragile or non-replicable (Haibe-Kains et al., 2020). These concerns are now being translated into governance instruments and standards: the NIST AI RMF frames trustworthiness as a lifecycle property (not a one-time claim), and its Generative AI Profile explicitly extends this stance to GenAI-specific risks and controls (National Institute of Standards and Technology, 2023; Autio et al., 2024). In parallel, risk-based regulation is hardening expectations around documentation, oversight, and risk management for high-impact systems (European Union, 2024).

The consequence is that trust has become the limiting reagent for real-world scientific and regulatory uptake. The key question is no longer whether agentic AI can produce plausible narratives, but whether it can produce claims and recommendations that remain auditable, reproducible in performance (under defined conditions), and explicit about uncertainty. Evidence-based disciplines offer a mature blueprint for this transition: evidence-based medicine was explicitly designed to replace persuasive narrative with protocolized, transparent evidence workflows (Sackett et al., 1996), and reporting standards such as PRISMA codify the expectation that evidence selection and synthesis be reproducible and inspectable (Page et al., 2021). The central premise of this perspective is that agentic AI is the first AI paradigm capable of *implementing* those evidence-based norms as executable infrastructure, turning “*trailblazing* (capability-first AI adoption that prioritizes performance and speed without commensurate governance or auditability)” capability into “*trustblazing* (evidence-based AI deployment that embeds provenance, reproducibility, and human accountability by design)” practice.

## 2 The evidence-based playbook: from EBM to EBT

Evidence-based medicine (EBM) emerged in response to two coupled problems: information overload (a rapidly expanding biomedical literature) and interpretive bias (the tendency for persuasive narrative and selective citation to dominate decision-making) (Hartung et al., 2025b). Its methodological pivot was not “*more papers*,” but more disciplined process: pre-specified questions, comprehensive and reproducible searches, explicit inclusion/exclusion rules, structured data extraction, and explicit appraisal of internal validity. The canonical formulation stresses that EBM is neither “*cookbook*” nor purely algorithmic: it is the integration of *individual clinical expertise* with the *best available external evidence* from systematic research (Sackett et al., 1996).

Over the last two decades, evidence-based toxicology (EBT) has translated these principles into toxicology’s uniquely heterogeneous evidence ecosystem, spanning human observational evidence, animal studies, *in vitro* systems, *in silico* models, and mechanistic reasoning (Hoffmann and Hartung, 2006; Hartung, 2009; Hoffmann et al., 2017; Hartung and Tsaioun, 2024). This translation was necessary because toxicological decision-making often depends on multiple evidence streams that differ in design, bias structures, endpoints, and inferential scope, and because traditional “*weight-of-evidence*” approaches (Linkov et al., 2015), while valuable, can become opaque when expert judgment is not operationalized with transparent criteria. A scoping review of guidance documents in toxicology and adjacent fields underscores the breadth (and unevenness) of available quality and reporting guidance across study types, and highlights the ongoing tension between assessing “*methodological quality*” broadly and focusing specifically on risk of bias (Hartung et al., 2025b) as the central threat to credibility (Samuel et al., 2016). Recent focus has been also on reporting problems and the need for standards (Percie du Sert et al., 2020; Mohapatra et al., 2025).

Across EBM and EBT, the core innovation is therefore traceable epistemology: making the pathway from question to evidence to inference to recommendation inspectable and reproducible. In practice, this playbook is increasingly standardized by interoperable components:

- Protocolized review and transparent reporting (e.g., PRISMA 2020)^1^ that document how studies were found, selected, appraised, and synthesized, enabling third-party audit and update (Page et al., 2021).
- Structured internal validity appraisal via risk-of-bias tools (e.g., RoB 2^2^ for randomized trials; ROBINS-I^3^ for non-randomized studies) (Hartung et al., 2025b) that replace informal “*study quality*” impressions with domain-based judgments and justification (Sterne et al., 2019; Sterne et al., 2016). Noteworthy, new tools are under development here (Mathisen et al., 2023; Mathisen et al., 2024; Mathisen et al., 2025; Svendsen et al., 2024; Vist et al., 2024; Bearth et al., 2025).
- Graded certainty (quality) of evidence and strength of recommendations, most prominently via GRADE,^4^ which makes explicit the domains that lower confidence (risk of bias, inconsistency, indirectness, imprecision, publication bias) and separates “*certainty*” from “*effect size*” (Guyatt et al., 2008).
- Evidence-to-Decision (EtD) frameworks that explicitly separate evidence appraisal from the value-laden step of turning evidence into recommendations, documenting trade-offs, feasibility, acceptability, and other decision criteria (Alonso-Coello et al., 2016). The Evidence-based Toxicology Collaboration (EBTC^5^) has a working group translating this to toxicology.
- Meta-review appraisal (e.g., AMSTAR 2)^6^ to assess whether systematic reviews themselves are methodologically fit for decision use (Shea et al., 2017).

EBT adapts these components to toxicology’s realities. Environmental and toxicological questions are often framed as PECO (Participants/Population, Exposure, Comparator, Outcome) rather than PICO (Intervention), and decision contexts frequently require integration of human and non-human evidence while preserving transparency about how streams were weighted. One influential operationalization is the Navigation Guide,^7^ which explicitly imports systematic review norms into environmental health while addressing departures from clinical hierarchies (e.g., the role of human observational evidence and the need to combine evidence streams) (Woodruff and Sutton, 2014). In parallel, governmental programs have institutionalized systematic review procedures for hazard and health assessments, notably the OHAT handbook (National Toxicology Program, 2019) and the U. S. EPA IRIS handbook process (U.S. Environmental Protection Agency, 2022), with independent scientific review of the IRIS procedures by the National Academies (National Academies of Sciences, Engineering, and Medicine, 2022).

Finally, as EBT becomes embedded in regulatory practice, risk-of-bias appraisal becomes the hinge between evidence abundance and evidence usability - particularly in domains where study design and reporting variability are high. Recent work has emphasized both the centrality of risk-of-bias to toxicological credibility and the emerging role (and limits) of AI in scaling bias detection without diluting methodological rigor (Hartung et al., 2025b). In this sense, EBM to EBT translation is not merely a methodological export; it is a necessary re-engineering of how toxicology can remain transparent, reproducible, and decision-relevant under modern evidence complexity.

## 3 Agentic AI as executable evidence infrastructure

Agentic AI is best understood not as a single model but as an orchestration pattern: specialized agents cooperate to execute multi-step, protocolized workflows, calling external tools (search, databases, calculators, code) while persisting intermediate artifacts (queries, retrieved passages, extracted fields, judgments, and decision tables). In toxicology and regulatory science this matters because many tasks are already workflow-shaped: evidence discovery, screening, extraction, risk-of-bias appraisal, mechanistic integration, and evidence-to-decision translation. In that sense, agentic AI can shift AI’s role from a passive “*knowledge-digesting algorithm on command*” to an orchestrator of active inquiry and self-optimization, with potential for self-directed workflows spanning literature review through study design and experimental optimization (Luechtefeld and Hartung, 2025).

Foundational “*reason-and-act*” prompting patterns demonstrate why orchestration is a genuine capability jump. ReAct explicitly interleaves reasoning traces with actions that query external sources (e.g., a Wikipedia API) and reports improved robustness over purely “*reasoning-only*” prompting by letting the model check itself against the environment (Yao et al., 2022). Complementarily, tool-use training approaches such as Toolformer show that language models can learn, in a largely self-supervised manner, to decide *which tool to call*, *when*, and *how to incorporate results*, a pathway to making “*verification*” an architectural default rather than a *post hoc* user behavior (Schick et al., 2023). Multi-agent frameworks then extend this logic to teams: a system can separate roles (retriever, extractor, critic, statistician, report-writer), with explicit handoffs and cross-checks; AutoGen provides an influential template for constructing such multi-agent conversations with tools and optional human inputs (Wu et al., 2023).

Retrieval-augmented generation (RAG) is the complementary infrastructure layer that addresses a core scientific requirement: ground outputs in citable source passages. The original RAG formulation combines parametric generation with non-parametric retrieval and motivates the approach explicitly in terms of provenance, inspectability, and updatability of knowledge (Lewis et al., 2020). Subsequent surveys underline both the rapid evolution of RAG and persistent technical hazards (retrieval failures, chunking artifacts, bias, scalability), reinforcing that “*grounding*” is an engineering and governance problem, not a slogan (Gao et al., 2023; Gupta et al., 2024). For high-stakes evidence work, the practical translation is an audit trail: what was retrieved, what was extracted, what was inferred, and what remains uncertain—turning narrative into inspectable evidence artifacts (Hartung, 2026).

Early demonstrations illustrate what “agentic + RAG” can look like in biomedicine. TxAgent integrates multi-step reasoning with real-time knowledge grounding across a large “*tool universe*” of 211 tools (Gao et al., 2025). In pharmacovigilance, MALADE^8^ orchestrates LLM-powered agents with RAG for adverse drug event extraction and reports strong performance against an OMOP ground truth table (AUC 0.90) while explicitly producing structured associations and explanations (Choi et al., 2024). These exemplars are not direct templates for regulatory toxicology, but they concretely show the direction: agents can decompose tasks, retrieve evidence, execute tool calls, and return structured outputs, i.e., precisely the ingredients needed for evidence-based workflows.

However, the point of “*executable evidence infrastructure*” is not autonomy; it is standardization at scale under meaningful oversight. Two limitations are especially salient for regulatory contexts. First, multi-step systems suffer from error compounding across long chains and face persistent challenges in uncertainty quantification and task-specific calibration (Luechtefeld and Hartung, 2025). Second, agentic AI for evidence synthesis remains an emerging trend with limited published evaluation of accuracy and safety; the evidence-synthesis community has already seen how rapidly adoption can outrun reporting and evaluation discipline (Schmidt et al., 2025). Consistent with regulatory discussions of AI adoption, the appropriate endpoint is therefore a “*co-pilot*” model: agents execute protocolized steps, but accountability, adjudication of conflicts, and final decisions remain with trained experts and pre-specified governance.

In sum, agentic AI becomes “*executable evidence infrastructure*” when it is engineered to (i) decompose evidence-based workflows into auditable steps, (ii) ground every claim in retrievable, citable sources, (iii) preserve intermediate artifacts for inspection and preserve human oversight structures that explicitly manage uncertainty, disagreement, and responsibility. That, not autonomous decision-making, is the trust-preserving promise of agentic systems in high-stakes science.

## 4 The evidence-based agent stack

**Figure 1:**
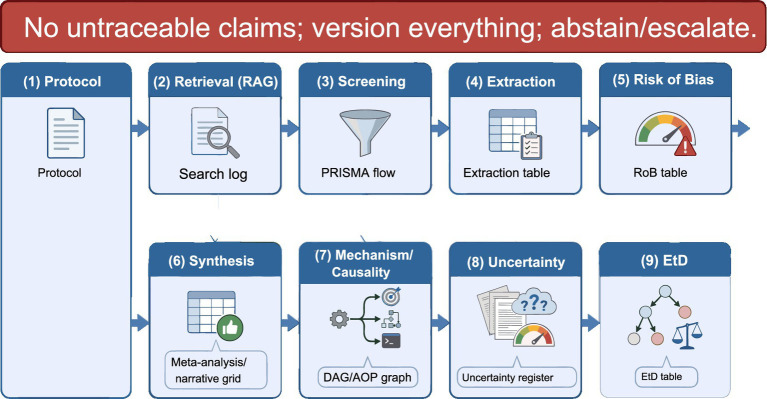
The evidence-based agent stack (workflow architecture). Evidence-based Agent Stack decomposes evidence workflows into narrowly mandated agents that produce structured, provenance-linked, versioned artifacts, mirroring systematic review and EtD panels while making the process executable and repeatable. Based on a figure generated with Figurelabs.ai.

To operationalize evidence-based AI in high-stakes settings, we propose an Evidence-based Agent Stack (Figure 1 and Box 1): a set of narrowly mandated agents whose outputs are structured, provenance-linked, and versioned. This decomposition mirrors how systematic review teams and evidence-to-decision panels actually work, i.e., separating roles (question framing, retrieval, screening, extraction, appraisal, synthesis, decision translation), but makes the workflow executable, repeatable, and auditable. In practice, each agent produces a defined artifact (protocol, search log, PRISMA flow, extraction table, risk-of-bias table, synthesis model, causal graph, uncertainty register, EtD table) that can be inspected by humans and reused by downstream agents. The key shift is that “*good scientific practice*” becomes a pipeline contract: agents are allowed to proceed only when upstream artifacts meet pre-specified criteria (e.g., protocol locked before screening; extraction fields marked “*not reported*” where absent; every quantitative value traceable to a source span). This logic operationalizes transparency expectations codified in systematic review reporting (PRISMA 2020) and aligns with the broader movement toward lifecycle trust and documentation in AI governance (Page et al., 2021; National Institute of Standards and Technology, 2023).

A RAG-first retrieval layer provides the epistemic substrate: agents are constrained to generate findings from retrieved, citable passages rather than model memory, supporting updating as knowledge changes and enabling auditability (Lewis et al., 2020). An existence proof of “*systematic evidence review as software*” is Sysrev,^9^ a Findable, Accessible, Interoperable, and Reusable (FAIR)^10^-oriented platform that operationalizes systematic evidence review and data curation via structured labeling, duplicate review, conflict resolution, and active-learning prioritization, exactly the kind of governance primitives that agentic evidence pipelines require (Bozada et al., 2021). Downstream, risk-of-bias appraisal becomes a hinge for credibility: automated assistance can improve scalability, but only if judgments remain justified and transparent, consistent with modern bias frameworks (e.g., RoB 2, ROBINS-I) and domain-specific toxicology adaptations (Sterne et al., 2019; Sterne et al., 2016; Hartung et al., 2025b). Critically, “*AI help*” here should be understood as structured drafting + consistency checking + evidence linking, not autonomous verdicts, because RoB remains a context-sensitive interpretive task and is a frequent source of downstream overconfidence if treated as a checkbox.

Two additional agents address where many evidence workflows fail: causality/mechanism integration and uncertainty handling (Hartung et al., 2026). Mechanistic reasoning increasingly relies on explicit causal representations - directed acyclic graphs (DAGs) (Pearl, 2009) and network models such as adverse outcome pathway (AOP) networks (Leist et al., 2017), because complex, networked mechanisms require operational causal toolboxes beyond informal “*mechanistic plausibility*” narratives (Pearl, 2009; Hernán and Robins, 2020; OECD, 2018). Uncertainty, meanwhile, must be elevated from footnote to first-class output: regulatory science explicitly expects uncertainty analysis to be systematic, documented, and decision-relevant (EFSA Scientific Committee, 2018). Finally, the Evidence-to-Decision agent anchors the “*last mile*” where trust often collapses: it translates graded certainty into recommendations using explicit EtD logic (e.g., GRADE EtD), ensuring the leap from evidence to action remains inspectable rather than rhetorical (Alonso-Coello et al., 2016).

BOX 1 **The Evidence-based Agent Stack: roles, outputs, and audit outputs.**

*Purpose:* A modular, protocolized multi-agent architecture that operationalizes evidence-based practice as executable infrastructure. Each agent has a narrow mandate and must emit structured, provenance-linked, versioned outputs before downstream steps can proceed.

1. Protocol agent (question to protocol lock)
  *Mandate:* Convert the initiating question into PECO/PICO specifications; define populations/participants, exposure or intervention, comparators, outcomes, eligible study designs, and analysis plan; preregister/lock protocol before screening begins.
  *Required artifacts:* Protocol document; eligibility criteria table; outcome hierarchy; analysis plan; deviations log.
  *Standards anchor:* PRISMA 2020 transparency expectations for review conduct and reporting (Page et al., 2021).
2. Retrieval agent (RAG-first evidence acquisition)
  *Mandate:* Retrieve evidence only from pre-defined corpora; return provenance-linked passages with stable identifiers; maintain search strings and retrieval settings.
  *Required artifacts:* Search log (queries, dates, sources); retrieved passage set; de-duplication report; corpus/version record.
  *Standards anchor:* Retrieval-augmented generation to ground outputs in citable sources (Lewis et al., 2020).
3. Screening agent (transparent selection)
  *Mandate:* Apply inclusion/exclusion criteria conservatively; document reasons for exclusion; manage dual-screening where configured; support conflict resolution escalations.
  *Required outputs:* PRISMA-style flow numbers; inclusion/exclusion log; conflict list; adjudication notes.
  *Standards anchor:* PRISMA 2020 study selection reporting (Page et al., 2021).
4. Extraction agent (schema-first data capture)
  *Mandate:* Extract predefined fields into structured schemas (dose/exposure metrics, duration, endpoints, statistics, covariates, model system details); never infer missing values, explicitly mark “*not reported*.”
  *Required artifacts:* Extraction table; field-level provenance links; data dictionary; unit normalization log.
5. Risk-of-bias agent (internal validity appraisal)
  *Mandate:* Apply domain-appropriate RoB frameworks; generate justified judgments with evidence anchors; flag reporting gaps; propose sensitivity analyses.
  *Required artifacts:* RoB table with domain judgments + rationales; provenance links; sensitivity analysis plan.
  *Standards anchors:* RoB 2 and ROBINS-I (Sterne et al., 2019; Sterne et al., 2016); toxicology-specific RoB emphasis and AI-assist opportunities (Hartung et al., 2025b); automation precedent (Marshall et al., 2016).
6. Synthesis agent (quantitative synthesis where justified)
  *Mandate:* Conduct meta-analysis when heterogeneity and design allow; otherwise produce structured narrative synthesis; separate descriptive findings from inferences and label assumptions.
  *Required outputs:* Effect size table; model specifications; heterogeneity diagnostics; narrative synthesis grid; reproducible code/parameters if applicable.
7. Mechanism/causality agent (explicit causal representation)
  *Mandate:* Build explicit causal models (e.g., DAGs) and/or AOP network representations; map evidence to causal links; integrate mechanistic and difference-making evidence streams.
  *Required outputs:* Causal graph(s) + assumptions; mapping of evidence to edges; alternative model set; counterfactual queries or identification notes.
  *Standards anchors:* Causal inference foundations (Pearl, 2009; Hernán and Robins, 2020); AOP framework and networks (OECD, 2018).
8. Uncertainty agent (uncertainty as an output, not a caveat)
  *Mandate:* Quantify and communicate uncertainty; propagate study limitations, indirectness, and model assumptions; produce calibrated uncertainty statements and scenario bounds.
  *Required outputs:* Uncertainty register; domain-wise uncertainty ratings; propagation notes; decision-relevant uncertainty summary.
  *Standards anchor:* Systematic uncertainty analysis in scientific assessment (EFSA Scientific Committee, 2018).
9. Evidence-to-decision agent (transparent translation to recommendations)
  *Mandate:* Translate graded certainty into recommendations via explicit EtD logic; document trade-offs (benefits/harms, feasibility, acceptability, equity, values); preserve dissent and rationale.
  *Required outputs:* EtD table; recommendation statement + certainty; trade-off ledger; dissent log.
  *Standards anchor:* GRADE EtD frameworks (Alonso-Coello et al., 2016).

*Cross-cutting controls (apply to all agents):*

- *Provenance:* Every extracted fact and numerical value must link to a retrieved source span (Lewis et al., 2020).
- *Versioning:* Model versions, prompts, schemas, corpora, and tool configurations are version-pinned (National Institute of Standards and Technology, 2023).
- *Escalation:* Any unresolved conflicts, low-confidence steps, or missing critical fields trigger human adjudication.
- *Separation of extraction vs inference:* Agents must label inferences explicitly and preserve raw extracted evidence as the auditable substrate.

## 5 Non-negotiables: provenance, versioning, accountability

Evidence-based agentic AI must enforce hard constraints that convert narrative fluency into scientific reliability. In toxicology and regulatory science, these constraints are not “*best practices*” but minimum governance requirements, because an LLM that produces persuasive, untestable narratives is operationally dangerous when outputs feed hazard conclusions, risk assessments, or policy decisions. The non-negotiables below define what it means for an agentic system to behave like an evidence workflow rather than a storytelling machine.

### 5.1 First: no untraceable claims (provenance as a chain of custody)

The core rule is simple: *every extracted fact, especially numeric values, must be linked to an exact source span*, and whenever a system transitions from extraction to normalization, interpretation, or synthesis, that transition must be explicit; inferred content must be labeled as inference rather than presented as extracted evidence. This discipline is not cosmetic: it converts an LLM output from “*convincing text*” into an auditable scientific output by preserving an inspectable chain of custody from retrieved evidence to extracted fields to downstream summaries. Practically, this implies that provenance is not just “*citations in the narrative,*” but machine-actionable linkage: each claim points to span IDs; retrieval logs and corpus snapshots are stored; and conflicting extractions are surfaced side-by-side rather than resolved by the model into false certainty. In the evidence-synthesis context, this mirrors long-standing demands for transparency in study identification and selection (e.g., PRISMA), but extends them into the agentic layer: if an agent cannot point to its evidence, its output is *out of scope* for high-stakes use.

### 5.2 Second: version everything (because “*pipeline drift*” is a scientific confounder)

Modern agentic pipelines are composite systems: model weights, prompts, retrieval index settings, chunking rules, extraction schemas, and post-processing logic all shape the final output. Small changes in any of these can shift results materially; without version pinning, you cannot distinguish genuine evidence updates from silent pipeline drift. The practical requirement is therefore to record, for every run, the exact model snapshot, prompt template, corpus/index version, retrieval configuration, schema versions, and run date. Consistent with this, Sysrev’s “*project cloning*” feature highlights why reproducibility must include preserved workflow configuration (templates/labels/structures), not only narrative reporting (Bozada et al., 2021). This aligns with broader reproducibility arguments in AI, namely that results are inseparable from pipeline specification and documentation, and that opacity around data and process undermines scientific trust (Haibe-Kains et al., 2020). It also motivates standardized documentation artifacts such as Model Cards (Mitchell et al., 2019) and Datasheets for Datasets (Gebru et al., 2021), which provide structured reporting of intended use, limitations, evaluation, and provenance, precisely the information needed to judge whether a tool is appropriate for a given regulatory context.

### 5.3 Third: evaluate in a declared context of use (fitness-for-purpose is not optional)

“*Works well*” is meaningless without an explicit task definition, acceptable error profile, and domain boundary; screening workflows may need very high recall, numeric extraction may require tight tolerances, and mechanistic relation extraction may tolerate uncertainty only if it is flagged and reviewable. Accordingly, evaluation must be done on held-out corpora, with prompt development separated cleanly from testing to prevent inadvertent overfitting and leakage. This is not a hypothetical risk: prompt tuning can be iterated until it looks excellent on a convenient subset, then silently degrade when applied to different reporting styles or study designs, an overfitting analogue that can inflate perceived performance. Recent evidence-synthesis work similarly emphasizes the need for transparent reporting of LLM-assisted methods and notes a tendency toward incomplete reporting in LLM papers (dataset splitting, prompt development, model choice, validation metrics), motivating structured evaluation templates for LLM-based screening and extraction. The implication for evidence-based agentic AI is that evaluation should be pre-registered where possible, externally validated, and reported with task-relevant metrics, consistent with the wider reproducibility agenda in AI (Haibe-Kains et al., 2020).

### 5.4 Fourth: quantify uncertainty and surface disagreement (forced clarity is a failure mode)

Toxicologic evidence is heterogeneous, sometimes contradictory, and often indirect; an agentic system that compresses this into a single clean answer can mislead users by hiding contestation and uncertainty. Evidence-grade outputs should therefore include calibrated confidence or uncertainty estimates, show conflicting sources with provenance, and allow abstention when evidence does not support reliable extraction. This corresponds to regulatory expectations that uncertainty analysis is central to scientific assessment rather than ancillary (EFSA Scientific Committee, 2018), and it becomes even more important in agentic workflows where downstream steps can be triggered automatically. A defensible operational default is thus: uncertainty is an output object (an “*uncertainty register*”), not a caveat paragraph.

### 5.5 Finally: accountability remains human (co-pilot, not autopilot)

Even a well-governed agentic stack does not eliminate responsibility; it relocates it. The governance principle is explicit: keep humans in the loop where accountability sits; apply risk-tiered human review, define escalation rules for high-impact outputs, and document adjudications and overrides so that corrections are learnable and audit-ready. This aligns with regulatory discussions advocating a gradual “*co-pilot*” approach in which AI augments human expertise while accountability frameworks define responsibilities and error-handling guidelines. In practical terms, “*meaningful human oversight*” must be operationalized as workflow gates (e.g., protocol lock before screening; adjudication before EtD translation), sign-off roles, and traceable decision logs, because, as emphasized in regulatory contexts, agencies and expert panels will likely retain ultimate responsibility for AI-assisted processes and outputs.

**Figure 2:**
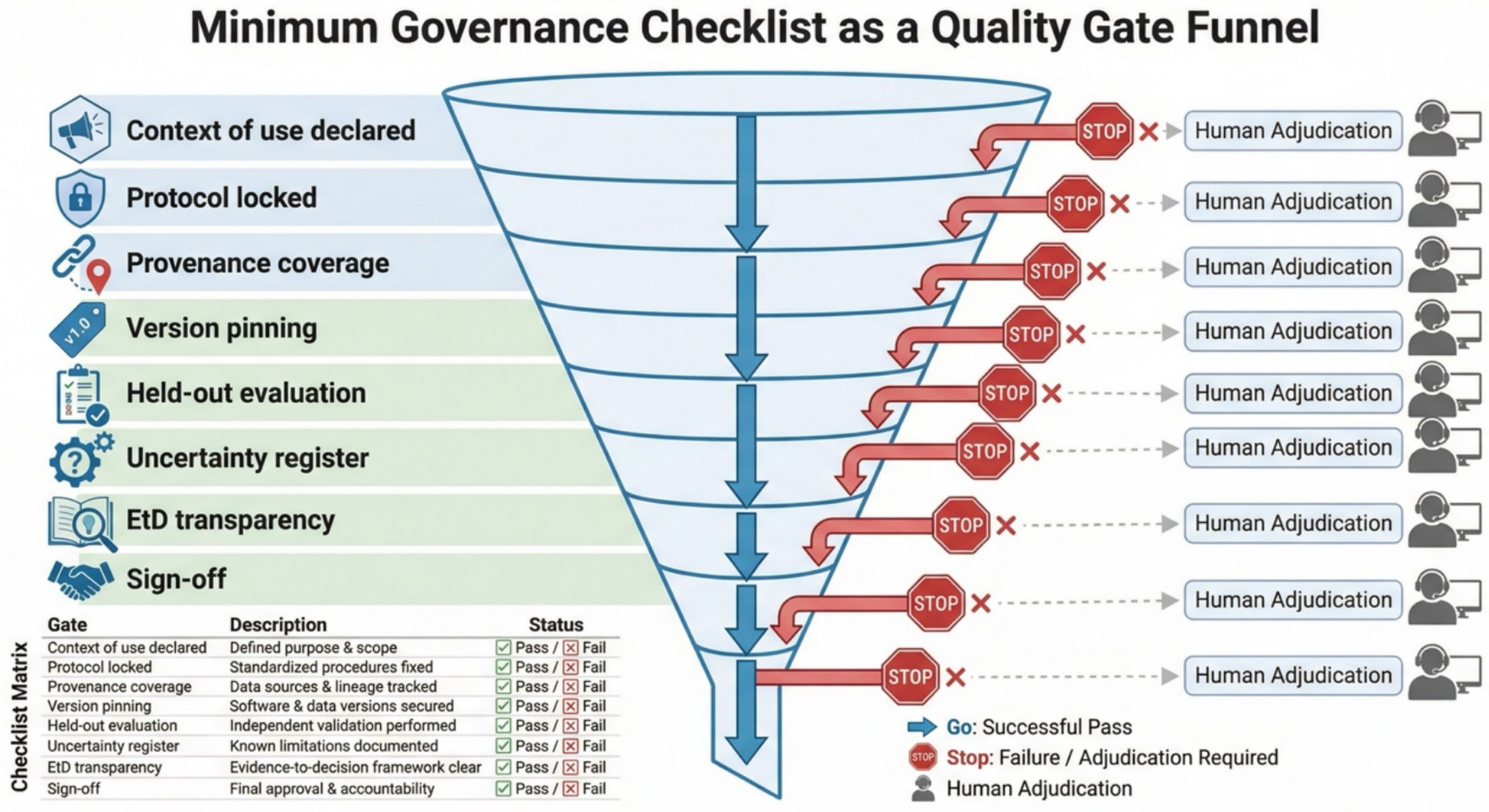
Minimum governance checklist as a “quality gate” funnel. Minimum governance gates transform narrative fluency into scientific reliability by enforcing provenance, versioning, evaluation discipline, uncertainty reporting, and accountability at predefined checkpoints. Based on a figure generated with Figurelabs.ai.

Taken together, these non-negotiables define evidence-based AI as governance-by-design: provenance and audit trails prevent black-box evidence; versioning prevents silent drift; context-of-use evaluation prevents inflated performance claims; uncertainty handling prevents false clarity; and accountability rules ensure that agency, responsibility, and legitimacy remain where they must, i.e., in expert human decision-making. Table 1 shows a suggested Minimum Governance Checklist for Evidence-Based Agentic AI (context-of-use ready) also illustrated as Figure 2.

**Table 1:** Minimum governance checklist for evidence-based agentic AI.

Governance control	Applies to (agent/pipeline step)	Minimum requirement (go/no-go)	Required artifacts (audit-ready)	Simple checks / metrics
1. Context of use declared	Whole system	Intended use, decision consequence, and risk tier explicitly stated before any run	Context-of-use statement; scope/exclusions; user roles	Context of Use (CoU) signed; risk tier assigned; out-of-scope guardrails enabled
2. Protocol locked before screening	Protocol + Screening	PECO/PICO defined; inclusion/exclusion and outcomes pre-specified; deviations tracked	Protocol; outcome hierarchy; deviations log; PRISMA flow template	Protocol timestamp < screening timestamp; deviation count reported
3. Provenance coverage	Retrieval → Extraction → Synthesis	No untraceable claims: every extracted fact/value linked to source span; inferences labeled	Retrieval log; span IDs; extraction table with span links; inference flags	≥95–100% of extracted numeric fields have span links; “*inference*” tags present
4. Corpus and retrieval determinism	Retrieval (RAG-first)	Predefined corpora only; retrieval config pinned; de-duplication recorded	Corpus snapshot/version; search strings; index hash; de-dup report	Re-run yields same retrieved set (or documented drift); retrieval parameters immutable per run
5. Version pinning	Whole system	Model, prompts, schemas, tools, and post-processing versions recorded	Model ID; prompt template ID; schema version; tool versions; run manifest	Manifest completeness = 100%; diffable run-to-run; change log present
6. Data extraction integrity	Extraction	Missing data recorded as “*not reported*”; units normalized with trace	Extraction schema + dictionary; unit normalization log; QC flags	Missingness rate reported; unit conversions reproducible; random sample QC pass
7. Risk-of-bias justification	RoB appraisal	RoB judgments must be domain-based and justified with evidence anchors	RoB table + rationales; supporting spans; conflict/adjudication notes	≥90–100% RoB fields have justification + span; disagreements surfaced
8. Separation of description vs. inference	Synthesis + Mechanism	Descriptive findings separated from causal/inferential statements; assumptions explicit	Synthesis report with labeled sections; assumptions register; causal model notes	“*Inference*” section present; assumptions enumerated; no causal claims without model
9. Uncertainty register mandatory	Uncertainty + EtD	Uncertainty quantified/graded and propagated to conclusions; abstention allowed	Uncertainty register; sensitivity analyses; confidence statements	Uncertainty attached to each key conclusion; “*unable to conclude*” permitted
10. Evidence-to-Decision transparency	EtD	Recommendation traceable to certainty + trade-offs; dissent preserved	EtD table; trade-off ledger; dissent log; final recommendation	Every recommendation has linked certainty + rationale; dissent rate reported
11. Human oversight gates	Whole system	Predefined sign-off points for high-impact outputs; escalation rules for conflicts	Review checklist; sign-off log; escalation criteria	Gate pass/fail recorded; % outputs requiring escalation tracked
12. External validation and reporting	Whole system	Held-out evaluation in stated CoU; transparent reporting of methods/limits	Evaluation protocol; test set description; metrics; failure analysis	Task-relevant metrics (e.g., recall for screening; exact-match for numerics); error taxonomy
13. Monitoring and re-validation triggers	Post-deployment	Drift detection; trigger thresholds for re-run/re-validation	Monitoring dashboard; drift report; re-validation plan	Drift thresholds defined; time-to-revalidate tracked
14. Security and access control	Whole system	Data governance (PHI/PII, i.e., Patient Health Information / Personally Identifiable Information), least privilege, audit logs	Access policy; audit logs; redaction policy	Audit log completeness; access reviews scheduled; leakage tests for prompts/logs

How to use: treat each row as a “*go/no-go*” gate. For low-risk exploratory use, you may relax thresholds; for regulatory decision support, keep them strict and auditable.

Standards anchors (examples): PRISMA 2020 for transparent review reporting; RAG for provenance grounding; NIST AI RMF for lifecycle risk management; EFSA uncertainty guidance; and reproducibility/documentation norms in AI (Page et al., 2021; Lewis et al., 2020; National Institute of Standards and Technology, 2023; EFSA Scientific Committee, 2018; Haibe-Kains et al., 2020; Mitchell et al., 2019).

## 6 Regulatory guardrails: TREAT and e-validation

Recent regulatory-science discussions propose TREAT (Trustworthiness, Reproducibility, Explainability, Applicability, Transparency) as a practical organizing principle for qualifying AI systems in regulatory settings (Hartung et al., 2025a). In this framing, “*trust*” is not treated as a vague aspiration but as a set of testable properties that can be negotiated against context-of-use constraints. For example, applicability (domain boundaries) is a familiar qualifier in biomarker and tool qualification, but the TREAT discussion explicitly surfaces tensions that matter for generative and agentic systems: whether rigid domain boundaries should be balanced with adaptability and monitoring; whether explainability is required even when performance appears strong; and how transparency should be balanced against innovation and proprietary constraints. These debates are not academic, regulators increasingly anticipate that AI will augment decision-making while not replacing human expertise, implying that oversight, interpretability (where needed), and documentation become prerequisites for adoption rather than optional virtues.

TREAT also makes explicit that several long-standing scientific concepts must be reinterpreted for AI. “*Reproducibility*” in traditional toxicology validation has often meant repeating the same protocol and obtaining comparable outcomes; however, deep learning and probabilistic systems can exhibit stochasticity, and multiple models can achieve similar performance with different internal features, raising the question of what should count as reproducible for regulatory purposes. In that sense, TREAT implicitly pushes validation from output replication to performance-centric reproducibility: consistent behavior under defined conditions, with quantified uncertainty and clear operational boundaries. This position is consistent with broader calls for transparency and reproducibility in AI research and for explicit lifecycle risk management (Haibe-Kains et al., 2020; National Institute of Standards and Technology, 2023).

However, the dynamic nature of modern AI challenges defies one-time “*validate-and-freeze*” paradigms (Hartung, 2024; Hartung and Kleinstreuer, 2025). In toxicology, classical *in vitro* method validation frequently takes years, an approach increasingly incompatible with rapidly evolving models, corpora, and prompting strategies, especially for NLP pipelines where updates to model versions, retrieval indices, or prompt templates can shift outputs materially. A lifecycle model is therefore emerging, in which continuing credibility maintenance rather than a single gatekeeping event is implemented. The *e-validation* framework (Hartung et al., 2024) was introduced precisely to reimagine validation “*through the lens of AI, translational science, and mechanistic relevance,*” and to address bottlenecks shared with other domains such as radiology, genomics, and medical-device software. At its core, e-validation explicitly proposes (Hartung and Kleinstreuer, 2025) a paradigm change from “*validate and forget*” to “*validate, monitor, and evolve,*” using real-world performance monitoring, back-testing as new data accumulate, and clear triggers for retraining or re-validation.

Crucially for this perspective’s thesis, e-validation represents a competing philosophy to TREAT, but as a way to operationalize TREAT across the lifecycle. Luechtefeld and Hartung (2025) state directly that the e-validation framework “*resonates with and operationalizes*” TREAT and reframes validation as a dynamic, evidence-responsive process rather than a one-time decision (Table 2). This alignment is highly pragmatic: TREAT supplies the *process*, including monitoring infrastructure, change-control expectations, and periodic requalification. It also dovetails with other regulatory trends toward modular, fit-for-purpose validation and evidence-weighted performance metrics (including confidence intervals) rather than binary concordance with legacy comparators.

**Table 2:** AI governance framework matrix: TREAT mapped to system properties and lifecycle (e-validation).

TREAT Principle	Design	Validation	Deployment	Monitoring	Re-validation
Trustworthiness	Protocol pre-registration, risk-of-bias assessment	External validation sets, blinded evaluation	Access controls, security audits	Performance monitoring, adverse event reporting	Change-control plans, impact analysis
Reproducibility	Environment versioning, code repository	Run manifests, independent verification	Containerization, deployment scripts	Operational logs, system state capture	Regression testing, automated Re-runs
Explainability	Feature importance analysis, model documentation	Interpretability methods (SHAP/LIME), error analysis	Model card for release, user guides	Real-time explanations, feedback loops	Updated explanations, rationale for changes
Applicability	Target population definition, data representativeness check	Subgroup analysis, generalizability tests	Integration testing, user training	Drift thresholds, usage metrics	Domain adaptation assessment, re-calibration
Transparency	Provenance logging, data source declaration	Audit trail creation, methodology disclosure	System status dashboard, version history	Alert logs, anomaly detection reports	Audit trail archive, public reporting

TREAT provides qualification criteria; e-validation operationalizes them across the lifecycle through monitoring, drift detection, and re-validation triggers—supported by companion agents and preserved audit outcomes.

A further regulatory-strengthening feature of e-validations “*companion agents*” (Hartung and Kleinstreuer, 2025): post-validation autonomous systems designed to sustain credibility after deployment; the paper describes a “*companion post-validation AI agent*” that would retrieve newly available data, assess representativeness, monitor performance, initiate retraining when needed, and perform back-testing, flagging discrepancies and alerting prior users if updates could change earlier conclusions. This concept maps cleanly onto practices emerging in other fields, notably medical-device software and continuous-learning frameworks. For example, the FDA’s AI/ML Software as a Medical Device (SaMD) Action Plan^11^ (FDA, 2019, 2021) and related policy work emphasize the continuous-learning nature of these models and the need for predetermined change-control protocols, real-time monitoring, and transparency measures. These precedents strengthen the argument that “*evolving model oversight*” is not merely desirable; it is increasingly the expected regulatory stance for adaptive AI systems.

Finally, e-validation and TREAT must be situated within a widening landscape of governance instruments. The emergence of regulatory sandboxes as controlled environments allows to test AI solutions with minimal risk, especially for early use cases like information retrieval and synthesis, before they are embedded in consequential workflows. The EU AI Act^12^ introduces conformity, documentation, and transparency obligations for high-risk systems, and ISO/IEC 42001 (ISO 2023)^13^ provides an AI map that can help organizations implement lifecycle governance. Together, these developments suggest that the regulatory future of agentic AI will be shaped by a layered model: (i) principle sets (like validation processes, such as e-validation), and (iii) organizational management systems and legal frameworks that enforce documentation, monitoring, and accountability.

In this context, evidence-based agentic AI aligns naturally with the lifecycle framing because it treats provenance, versioning, and uncertainty reporting as built-in workflow properties, not as after-the-fact documentation. When an Evidence-based Agent Stack requires retrieval-grounded claims, preserved audit trails, and explicit uncertainty registers, it becomes inherently compatible with TREAT expectations; when it additionally pins versions and monitors drift, it becomes compatible with e-validation’s “*monitor and evolve*” obligation. The result is a credible pathway from trailblazing capability to trustblazing deployment: progressive adoption via sandboxes, escalating contexts of use only as TREAT criteria are met continuously under e-validation governance.

## 7 Avoiding automation traps: evaluation must scale with capability

**Figure 3:**
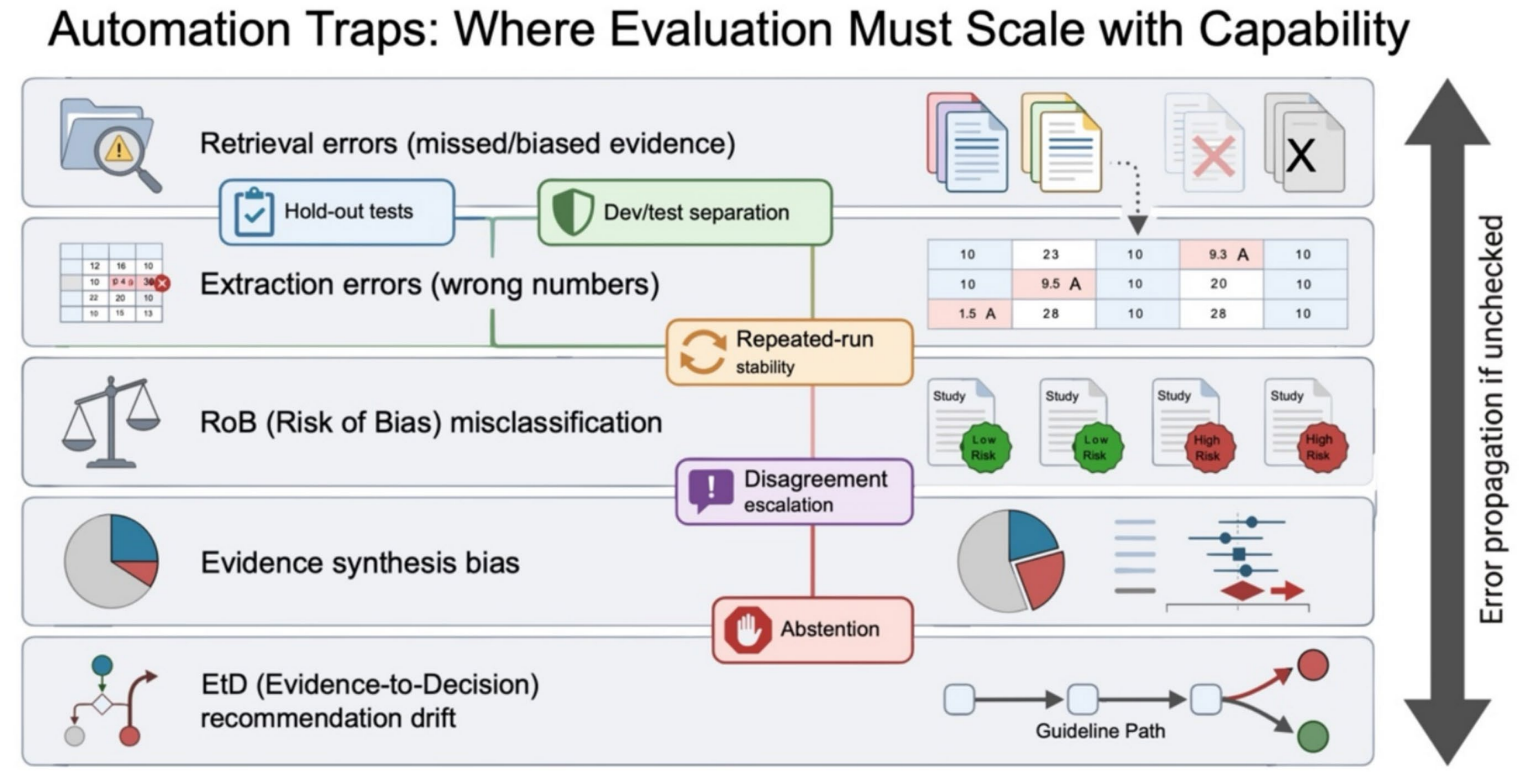
Automation traps: evaluation must scale with capability. As automation increases, evaluation must scale: weaknesses in retrieval, extraction, or bias appraisal can compound across agent chains. Evidence-based agentic AI embeds test separation, transparent reporting, and escalation pathways to prevent downstream overconfidence. Based on a figure generated with Figurelabs.ai.

Automation can *degrade* scientific standards when evaluation and reporting do not keep pace with rapidly expanding capability (Figure 3). LLM-assisted data extraction, anchored in a living systematic review of automated extraction methods, can document such patterns, i.e., as LLM use grows, gaps in evaluation standards, reproducibility, and fair benchmarking have emerged; adherence to established reporting best practices appears to be declining; and some papers report the counter-intuitive result that smaller (non-generative) models can outperform LLMs in domains with high-quality gold-standard datasets (Chen et al., 2025; Hu et al., 2024; Lu et al., 2024; Rehana et al., 2024; Schmidt et al., 2025). Complementarily, Sysrev shows how review automation can be coupled to governance controls (e.g., duplicate review, concordance requirements, and administrator-mediated conflict resolution), but these platform-level safeguards still depend on rigorous held-out evaluation and transparent reporting to prevent downstream error propagation (Bozada et al., 2021). The warning is methodological rather than ideological: without strong evaluation design, the field risks rewarding *impressive-looking demos* rather than reliable evidence infrastructure.

### 7.1 First, prompt engineering can masquerade as validation

Two factors drive these “*automation traps*” in practice. In LLM extraction, developers often iterate prompts interactively on small, convenient development sets, then inadvertently test on data that are too similar (or even overlapping) with those used in prompt tuning. This is a recurring failure mode: inflated performance from tiny evaluation sets, leakage between prompt development and testing, and sensitivity to dataset splits and reporting idiosyncrasies; prompts can be tuned to look excellent on a convenient subset and then silently degrade when applied to different study designs or endpoints. Schmidt et al. (2025) make the same point with concrete examples: evaluation results vary widely in small studies, and maintaining strictly separate data subsets for prompt development and evaluation is essential to avoid overestimation. In other words, the “*model*” is not just the weights; the *effective model* includes the prompt, schema constraints, retrieval settings, and post-processing and the evaluation must treat it that way.

### 7.2 Second, evaluation in evidence workflows is hard to automate and easy to mis-specify

Schmidt et al. (2025) note that LLM outputs (and accuracy) can show small changes between runs that may not be controllable via parameters, implying that evaluations may need to be repeated rather than treated as single-shot scores. They also highlight that gold-standard datasets enable sustainable re-training and re-evaluation *if* the community publishes reusable datasets and code; diversity of non-overlapping datasets is crucial to prevent overfitting to a single (possibly outdated) benchmark. At the same time, toxicology often lacks shared benchmarks for its most consequential tasks, i.e., structured extraction from toxicological studies, mechanistic relation mining, and risk-of-bias assessment, making performance claims difficult to compare across studies and systems. This is the classic “*evaluation debt*” problem: capability rises faster than the shared infrastructure for rigorous comparison.

These traps are intensified in agentic systems, where extraction outputs are not endpoints but inputs to downstream synthesis and decisions. Without explicit error handling, a minor extraction mistake can propagate into a seemingly coherent recommendation. This is why evidence-based agentic AI should embed evaluation and reporting controls *as pipeline gates*, not as optional appendices:

1. Held-out evaluation designed for the workflow (not a generic benchmark): define the task (screening vs. numeric extraction *vs* relationship extraction), acceptable error profiles, and decision consequences; evaluate on held-out corpora that reflect the stated context of use.
2. Strict separation of development from testing: treat prompt engineering, schema design, retrieval settings, and post-processing as part of the model; lock them before test evaluation.
3. Transparent reporting of prompts, datasets, and system versions: including model identifiers, retrieval corpora and indexes, chunking choices, schema versions, and sampling/temperature settings, so results can be audited and replicated in performance. Clinical AI has converged on reporting extensions for precisely this reason: CONSORT-AI^14^ (Liu et al., 2020) and SPIRIT-AI^15^ (Cruz Rivera et al., 2020) for AI interventions in trials, DECIDE-AI^16^ (Vasey et al., 2022) for early-stage evaluation, and TRIPOD+AI^17^ (Collins et al., 2024) for prediction models.
4. Explicit error-handling and disagreement escalation pathways: require abstention when provenance is missing, surface conflicts across sources, and route low-confidence or high-impact outputs to human adjudication rather than forcing a single “*best guess*.”

Two additional practices deserve emphasis for agentic evidence pipelines. (i) Repeated-run evaluation and stability reporting should become routine when model outputs vary run-to-run; report distributions (not just point estimates) and characterize variance under controlled settings. (ii) Cross-dataset generalization tests should be treated as a minimum bar, because performance often drops in more granular tasks and across domains; reliance on internal validation alone is widely recognized as insufficient when the goal is real-world deployment.

Conceptually, the message is the same as in “*hidden technical debt*” (Sculley et al., 2015) arguments from ML systems engineering: quick wins are real, but without disciplined evaluation, monitoring, and documentation, maintenance and reliability costs accumulate and failures emerge late, downstream, and expensively. In evidence-based toxicology, those “*downstream costs*” are not just technical, they are epistemic and regulatory. The remedy is not slower innovation; it is evaluation discipline that scales with capability.

## 8 Conclusion: becoming trustblazers

**Figure 4:**
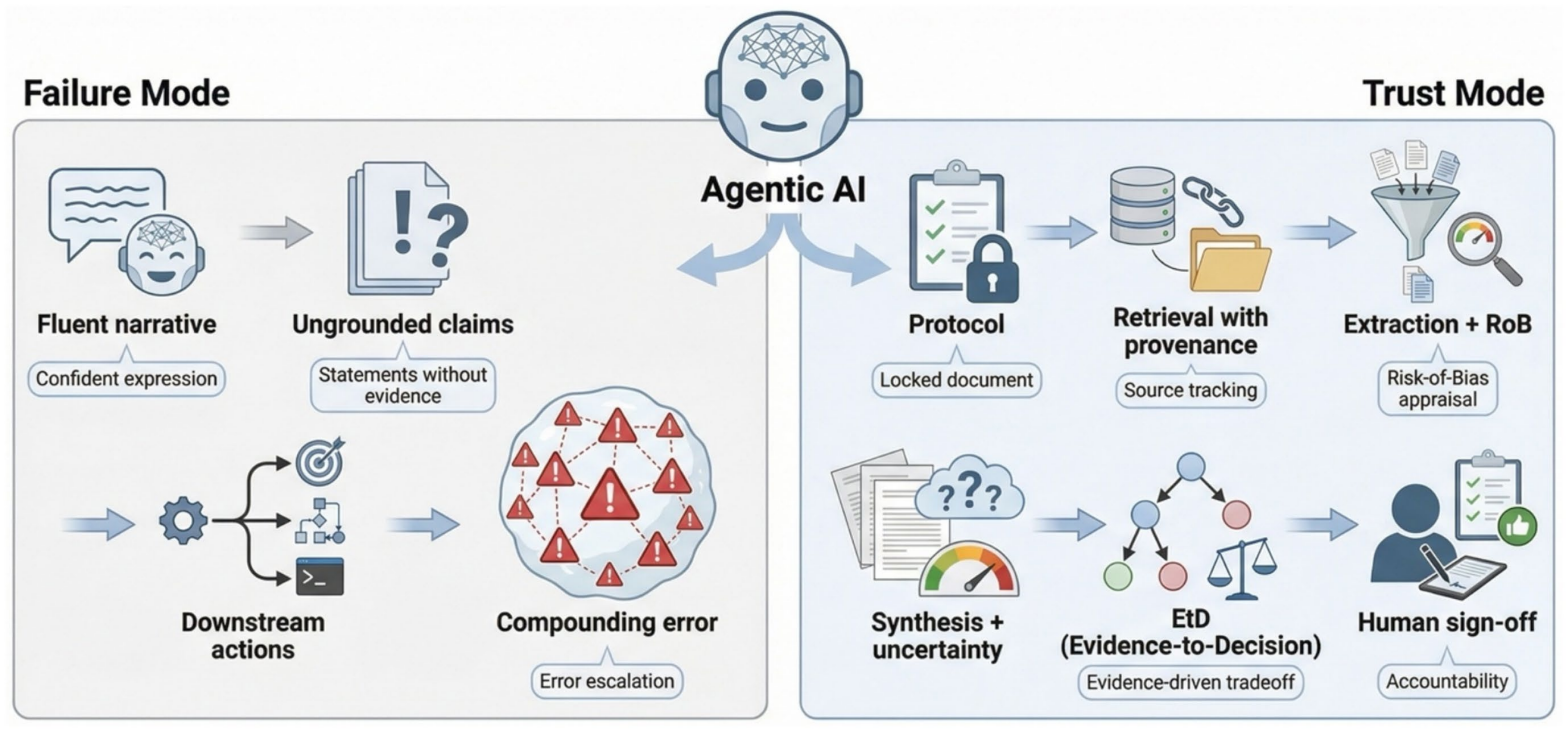
From trailblazer to trustblazer: two pathways for agentic AI. Agentic AI can amplify confident narrative or enable evidence-based decision support. Trustblazing requires executable constraints, i.e., protocol locking, provenance, versioning, risk-of-bias appraisal, uncertainty registers, and evidence-to-decision translation under human accountability. Figure generated with Figurelabs.ai.

Agentic AI is a fork in the road (Figure 4). It can either amplify the worst failure mode of modern information ecosystems (confident, ungrounded narrative optimized for persuasion) or become the enabling infrastructure of evidence-based science at scale. The difference will not be determined by model size or rhetoric, but by whether we treat trustworthiness as an *emergent property* of good intentions or as a set of engineered constraints: provenance requirements, versioned pipelines, context-of-use evaluation, uncertainty registers, and explicit human accountability.

The scientific community has navigated a structurally similar transition before. Evidence-based medicine (EBM) emerged because narrative authority could not keep pace with a growing literature and rising stakes; its central innovation was transparent, protocolized process rather than “*more evidence*.” The EBM playbook, i.e., pre-specified questions, reproducible retrieval, explicit inclusion/exclusion criteria, structured extraction, appraisal of internal validity, and graded certainty, remains one of the most successful governance mechanisms for transforming information into defensible decisions (Sackett et al., 1996; Page et al., 2021). Evidence-based toxicology (EBT) adapted these principles to a domain with even more heterogeneous evidence streams and inferential challenges, where mechanistic reasoning and cross-system translation must be integrated without obscuring uncertainty (Hoffmann et al., 2017; Hartung and Tsaioun, 2024).

This perspective’s core claim is that agentic AI is the first AI paradigm capable of implementing this playbook as executable infrastructure. With the Evidence-based Agent Stack, protocol discipline becomes a pipeline gate (protocol locked before screening); retrieval becomes provenance-bearing (RAG-first with span-linked claims); extraction becomes schema-driven (“*not reported*” rather than inferred); risk-of-bias appraisal becomes justified and auditable; synthesis becomes explicitly separated from inference; causality becomes graph-based and assumption-aware; uncertainty becomes an output object; and evidence-to-decision translation becomes transparent through structured EtD logic. In this form, “*evidence-based*” is not a label, it is software architecture.

Regulatory science offers a complementary lens. TREAT (Trustworthiness, Reproducibility, Explainability, Applicability, Transparency) provides a compact criterion set for qualifying AI in regulatory contexts (Hartung et al., 2025a). But it is increasingly clear that one-time “*validate-and-freeze*” approaches cannot keep pace with dynamic AI systems. E-validation reframes qualification as a lifecycle process “*validate, monitor, detect drift, and trigger re-validation*” potentially supported by companion agents that track new evidence and signal when conclusions may need revision (Luechtefeld and Hartung, 2025). Evidence-based agentic AI is naturally compatible with this lifecycle model because provenance, versioning, and uncertainty are built in as workflow properties, making monitoring and requalification tractable rather than aspirational.

Yet the road to trustblazing is not automatic. As emphasized in a toxicology-focused analyses of LLM-assisted extraction (Schmidt et al., 2025), automation can outpace evaluation norms; reporting can become thinner, and smaller models can outperform LLMs when robust gold standards exist, an empirical reminder that capability claims must be earned through rigorous, transparent evaluation. The remedy is to embed evaluation and governance in the pipeline itself: held-out testing; separation of development from testing; prompt and dataset transparency; error-handling and disagreement escalation; and explicit abstention when evidence does not support reliable conclusions.

What, then, does it mean to become “*trustblazers”*? It means accepting that the most important frontier is not autonomous decision-making, but auditable decision support. It means shifting from “*AI that can write*” to AI that can “*show its work*” with provenance-linked claims, versioned outputs, and uncertainty communicated in decision-relevant terms. It means treating trust as measurable and maintained: not a marketing property of a model, but a lifecycle property of a system embedded in human responsibility structures (National Institute of Standards and Technology, 2023).

If we encode evidence-based practice as executable constraints on agentic workflows, we can transform trailblazing AI into *trustblazing* AI: systems that are auditable, updateable, and aligned with human accountability, capable of scaling evidence synthesis and decision support without diluting the epistemic standards that make science reliable in the first place.

## Data Availability

The original contributions presented in the study are included in the article/supplementary material, further inquiries can be directed to the corresponding author.

## Footnotes

1
https://www.prisma-statement.org/prisma-2020

2
https://www.riskofbias.info/welcome/rob-2-0-tool

3
https://methods.cochrane.org/bias/risk-bias-non-randomized-studies-interventions

4
https://www.gradeworkinggroup.org

5
https://www.ebtox.org

6
https://amstar.ca/Amstar-2.php

7
https://prhe.ucsf.edu/navigation-guide

8
https://github.com/jihyechoi77/malade

9
https://www.sysrev.com

10
https://www.go-fair.org/fair-principles/

11
https://www.fda.gov/medical-devices/software-medical-device-samd/artificial-intelligence-software-medical-device

12
https://artificialintelligenceact.eu

13
https://www.iso.org/standard/42001

14
https://www.equator-network.org/reporting-guidelines/consort-artificial-intelligence/

15
https://www.equator-network.org/reporting-guidelines/spirit-artificial-intelligence/

16
https://www.equator-network.org/reporting-guidelines/reporting-guideline-for-the-early-stage-clinical-evaluation-of-decision-support-systems-driven-by-artificial-intelligence-decide-ai/

17
https://www.equator-network.org/reporting-guidelines/tripod-statement/
